# Expectations for methodology and translation of animal research: a survey of health care workers

**DOI:** 10.1186/s12910-015-0024-x

**Published:** 2015-05-07

**Authors:** Ari R Joffe, Meredith Bara, Natalie Anton, Nathan Nobis

**Affiliations:** Department of Pediatrics, University of Alberta, Edmonton Clinic Health Academy, 4-546 11405 87 Ave, Edmonton, AB Canada; John Dossetor Health Ethics Center, University of Alberta, Edmonton, AB Canada; Faculty of Medicine and Dentistry, University of Alberta, Edmonton, AB Canada; Department of Philosophy, Morehouse College, Atlanta, GA USA; Community Health & Preventive Medicine, Morehouse School of Medicine, Atlanta, GA USA

**Keywords:** Animal models, Animal research, Ethics, Methodology

## Abstract

**Background:**

Health care workers (HCW) often perform, promote, and advocate use of public funds for animal research (AR); therefore, an awareness of the empirical costs and benefits of animal research is an important issue for HCW. We aim to determine what health-care-workers consider should be acceptable standards of AR methodology and translation rate to humans.

**Methods:**

After development and validation, an e-mail survey was sent to all pediatricians and pediatric intensive care unit nurses and respiratory-therapists (RTs) affiliated with a Canadian University. We presented questions about demographics, methodology of AR, and expectations from AR. Responses of pediatricians and nurses/RTs were compared using Chi-square, with *P <* .05 considered significant.

**Results:**

Response rate was 44/114(39%) (pediatricians), and 69/120 (58%) (nurses/RTs). Asked about methodological quality, most respondents expect that: AR is done to high quality; costs and difficulty are not acceptable justifications for low quality; findings should be reproducible between laboratories and strains of the same species; and guidelines for AR funded with public money should be consistent with these expectations. Asked about benefits of AR, most thought that there are sometimes/often large benefits to humans from AR, and disagreed that “AR rarely produces benefit to humans.” Asked about expectations of translation to humans (of toxicity, carcinogenicity, teratogenicity, and treatment findings), most: expect translation >40% of the time; thought that misleading AR results should occur <21% of the time; and that if translation was to occur <20% of the time, they would be less supportive of AR. There were few differences between pediatricians and nurses/RTs.

**Conclusions:**

HCW have high expectations for the methodological quality of, and the translation rate to humans of findings from AR. These expectations are higher than the empirical data show having been achieved. Unless these areas of AR significantly improve, HCW support of AR may be tenuous.

## Background

Biomedical animal research (AR) involves some harm to sentient animals including distress (due to confinement, boredom, isolation, and fear), pain, and early death [1-3]. AR is said to be morally permissible because the balance of these costs (harms to the animals) and benefits (to human medical care, quality of life, and survival) is favorable [4]. It is generally assumed that the benefits are great to human medicine [5]. An awareness of the empirical costs and benefits of AR is an important issue in medicine for several reasons. Health care workers (HCW) often perform (and are expected to perform) AR, promote AR directly with trainees and indirectly as role models, and advocate for use of public funds (from granting agencies and charitable foundations) toward medical related AR.

There is a growing literature that raises concerns about the empirical practice of AR in at least two domains. First, the methodological quality of AR is often poor in both experimental design and animal welfare aspects [6-12]. AR publications rarely report the use of eligibility criteria, randomization, allocation concealment, blinding, sample size calculation, primary outcome specification, and study replication [6-10,13,14]. AR publications rarely report performing a systematic review to determine the necessity of the research project, rarely report the use of continuous monitoring of the level of anesthesia or pain control, and often do not report the use of acceptable methods of euthanasia [7,11,12]. Second, the translation rate from AR to humans has been disappointing [15-18]. Extensive AR in the fields of sepsis [19-21], stroke [22,23], spinal cord injury [24,25], traumatic brain injury [26], cancer [27,28], degenerative neurological diseases [29,30], acquired immunodeficiency syndrome [31], asthma [32], and other fields [15-18] has translated to humans in 0-5% of cases. Pharmaceutical companies have found that, of drugs that work well in AR and progress to human clinical trials, only ≤8% are found safe and effective enough for market approval [33,34]. AR to determine toxicology [17,35-37], carcinogenicity [17,37-39], and teratogenicity [17,40] of drugs or compounds is no more accurate than chance, with concordance rates between species generally <40%.

Since most AR is funded by public money through government and charitable granting agencies, it is important to know the public perception of, and the level of public support for AR. Surveys of the public find that the majority are ‘conditional acceptors’ of AR; they accept the practice because of the promise of cures and treatments for life-threatening and debilitating human diseases, so long as animal welfare is at least minimally considered and protected [41]. To our knowledge, no survey has asked for the details of this conditional acceptance of AR. In this survey we ask HCW directly what the minimal acceptable standards in AR methodology might be, and what the minimal acceptable translation rate of AR to human treatments might be. This is important in order to determine how strong the support is for the *empirical practice* of AR, and how AR could be improved to increase the level of support. We found that HCW have high expectations for the methodological quality of, and the translation rate to humans of findings from AR.

## Methods

### Questionnaire administration

All pediatricians and pediatric intensive care unit nurses and respiratory therapists (RTs) who are affiliated with one Canadian University were e-mailed the survey using an electronic, secure, survey distribution and collection system (REDCap, Research Electronic Data Capture) [42]. A cover letter stated that “we very much value your opinion on this important issue” and that the survey was anonymous and voluntary. We offered the incentive that if the response rate was at least 70% we would donate $1000 to the Against Malaria Foundation or the PICU Social Committee. Non-responders were sent the survey by e-mail at 3-week intervals for 3 additional mailings.

### Questionnaire development

We followed published recommendations [43]. To generate the items for the questionnaire, we searched Medline from 1980 to 2012 for articles about the methodology and translation of AR. This was followed by collaborative creation of the background section and questions for the survey by the authors. Content and construct validation were done using a table of specifications filled out by experts including two ethics philosophy professors, and two pediatricians. Face and content validation were done by pilot testing of the survey, by non-medical, university-educated lay people (n = 9), pediatricians (n = 2), pediatric intensive care nurses (n = 2), and an ethics professor (n = 1). Each pilot test was followed by a semi-structured interview by 1 of the authors to ensure clarity, realism, validity, and ease of completion. A published clinical sensibility tool was used for the expert and pilot testing [43]. After minor modifications, the survey was approved by all the authors.

### Questionnaire content

The background section stated: “In this survey, ‘animals’ means: mammals, such as mice, rats, dogs, and cats. It has been estimated that over 100 million animals are used in the world for research each year. There are many good reasons to justify AR, which is the topic of this survey. Nevertheless, some people argue that these animals are harmed in experimentation, because their welfare is worsened. In this survey, ‘harmful’ means such things as: pain, suffering (disease/injury, boredom, fear, confinement), and early death. This survey is about how AR should be performed. We value your opinion on the very important issue of the methodology of AR.”

We presented demographic questions, 15 questions that asked respondents “about the methods of AR that are commonly discussed by animal researchers”, 4 questions that asked the respondent “to consider what you think the benefits to humans are as a result of AR”, and 8 questions that asked the respondent “for your opinions about what you expect from AR paid for with public funds (for example, funding by government using tax dollars, or charitable foundations using donations).” Response choices included scales of “strongly agree, agree, undecided, disagree, strongly disagree”, “nearly always, often, sometimes, not often, almost never”, and “5-20%, 21-40%, 41-60%, 61-80%, over 80%” depending on the type of question. All the questions are shown in the Tables 1, 2, 3, and 4.

**Table 1:** **Demographics of respondents**

**Respondent group**	**Pediatricians (n = 48)**	**Nurses/RTs (n = 69)**
Age		
18-24 yr	-	4 (7%)
25-34 yr	3 (6%)	38 (55%)
35-44 yr	16 (33%)	15 (22%)
45-54 yr	17 (35%)	8 (12%)
>54 yr	12 (25%)	3 (4%)
Sex (Male)	26 (54%)	7 (10%)
AR Experience		
Have done AR experiments in past	25 (52%)	2 (3%)
Currently do AR	4 (8%)	0 (0%)
Have never done AR	19 (40%)	67 (97%)

**Table 2:** **Healthcare Worker expectations regarding the methodology used in Animal Research**

**Statement about methodology**	**Respondent group**	**Strongly agree**	**Agree**	**Uncertain**	**Disagree**	**Strongly disagree**
Anesthetic used should be monitored (to ensure a deep enough level of anesthetic) during surgery on an animal in an experiment.						
	Pediatricians	30/44 (68%)	14/44 (32%)	0	0	0
	Nurses/RTs	51/67 (76%)	15/67 (22%)	1/67 (2%)	0	0
Pain should be monitored after this surgery (to ensure comfort) so that adequate pain medications are given if the animal is in pain.						
	Pediatricians	29/44 (66%)	14/44 (32%)	1/44 (2%)	0	0
	Nurses/RTs	49/68 (72%)	18/68 (27%)	1/68 (2%)	0	0
Pain should be monitored after this surgery even over-night (to ensure comfort) so that adequate pain medications are given if the animal is in pain.						
	Pediatricians	26/44 (59%)	14/44 (32%)	3/44 (7%)	1/44 (2%)	0
	Nurses/RTs	49/68 (72%)	17/68 (25%)	1/68 (2%)	1/68 (2%)	0
When an animal is killed at the end of the experiment, it is acceptable to use a cheaper but less humane method of killing to reduce cost.						
	Pediatricians	0	2/44 (5%)	6/44 (14%)	10/44 (23%)	26/44 (59%)
	Nurses/RTs	0	2/68 (3%)	7/68 (10%)	14/68 (21%)	45/68 (66%)
When an animal is killed at the end of the experiment, it is acceptable to use a less humane method of killing if this will improve the experimental results.						
	Pediatricians	1/44 (2%)	9/44 (21%)	11/44 (25%)	11/44 (25%)	12/44 (27%)
	Nurses/RTs	1/68 (2%)	7/68 (10%)	5/68 (7%)	15/68 (22%)	40/68 (59%)
To save time and money, it is acceptable to do an animal experiment even if there is another way to look at the experimental question that would not require using animals.						
	Pediatricians	0	5/44 (11%)	7/44 (16%)	17/44 (39%)	15/44 (34%)
	Nurses/RTs	0	5/68 (7%)	3/68 (4%)	19/68 (28%)	41/68 (60%)
To save time and money, it is acceptable to do an animal experiment using more animals even if statistical experts know of another way to look at the experimental question that requires using fewer animals.						
	Pediatricians	0	3/44 (7%)	3/44 (7%)	19/ 44(43%)	19/44 (43%)
	Nurses/RTs	0	6/68 (9%)	1/69 (2%)	21/68 (31%)	40/68 (59%)
To save time and money, it is acceptable to do an animal experiment without doing a comprehensive literature review that would determine if the study has already been done by another research group.						
	Pediatricians	0	0	0	12/44 (27%)	32/44 (73%)
	Nurses/RTs	0	1/68 (2%)	1/68 (2%)	21/68 (31%)	45/68 (66%)
It is acceptable to follow a research method that gives less reliable results in order to avoid the added costs of consulting an expert in study design.						
	Pediatricians	0	3/44 (7%)	2/44 (5%)	10/44 (23%)	29/44 (66%)
	Nurses/RTs	0	0	2/68 (3%)	31/68 (46%)	35/68 (52%)
It is acceptable to follow a research method that gives less reliable results in order to avoid the added costs of hiring more laboratory staff.						
	Pediatricians	0	3/44 (7%)	5/44 (11%)	16/44 (36%)	20/44 (46%)
	Nurses/RTs	0	0	3/67 (5%)	31/67 (46%)	33/67 (49%)
If an animal model of a disease has consistently found that successful treatments in animals have failed to work in humans, it is still acceptable to use that animal model to test a different treatment for that disease.						
	Pediatricians	0	13/44 (30%)	12/44 (27%)	10/44 (23%)	9/44 (21%)
	Nurses/RTs	1/68 (2%)	17/68 (25%)	17/68 (25%)	18/68 (27%)	15/68 (22%)
Some animal care practices result in stressed animals, which can have effects on their immune system and behavior. It is still acceptable to use these animal care practices in research looking at how a new treatment affects the immune system and behavior.						
	Pediatricians	1/43 (2%)	15/43 (35%)	5/43 (12%)	12/43 (28%)	10/43 (23%)
	Nurses/RTs	2/68 (3%)	11/68 (16%)	13/68 (19%)	16/68 (24%)	26/68 (38%)
If several experimenters are involved in an animal research study, they should each have similar training so they perform the procedures and treatments consistently.						
	Pediatricians	19/42 (45%)	22/42 (52%)	0	0	1/42 (2%)
	Nurses/RTs	36/67 (54%)	29/67 (43%)	1/67 (2%)	0	1/67 (2%)
Some research methods are important to getting reliable results, such as: deciding the most important result to be looked for, choosing animals for each treatment at random, and measuring results without knowing which treatment each animal received. It is acceptable not to follow any of these methods because of the added costs.						
	Pediatricians	0	3/43 (7%)	0	15/43 (35%)	25/43 (58%)
	Nurses/RTs	2/68 (3%)	0	7/68 (10%)	18/68 (27%)	41/68 (60%)
For animal research paid for with public funds (for example, funding by government using tax dollars, or charitable foundations using donations), guidelines consistent with your answers above should be required.						
	Pediatricians	19/42 (45%)	21/42 (50%)	2/42 (5%)	0	0
	Nurses/RTs	32/68 (47%)	30/68 (44%)	5/68 (7%)	1/68 (2%)	0

There were no statistically significant differences in responses between pediatricians and nurses/RTs on any of these questions.

**Table 3:** **Healthcare Worker perception of the benefits to humans from animal research**

**Statement about possible benefits**						
	**Respondent group**	**Nearly always**	**Often**	**Sometimes**	**Not often**	**Almost never**
How often do you think that a treatment discovered in animal research works in humans (a direct benefits to humans)?						
	Pediatricians	0	12/43 (28%)	21/43 (49%)	7/43 (16%)	3/43 (7%)
	Nurses/RTs	0	22/68 (32%)	35/68 (52%)	10/68 (15%)	1/68 (2%)
How often do you think a discovery in animal research contributes to other evidence that later eventually leads to a treatment for humans (an indirect benefit to humans)?						
	Pediatricians	2/43 (5%)	20/43 (47%)	14/43 (33%)	7/43 (16%)	0
	Nurses/RTs	0	24/68 (35%)	36/68 (53%)	8/68 (12%)	0
Is it your impression that animal researchers claim to the public that there are large benefits to humans from their research?						
	Pediatricians	4/43 (9%)	18/43 (42%)	17/43 (40%)	4/43 (9%)	0
	Nurses/RTs	15/68 (22%)	34/68 (50%)	17/68 (25%)	2/68 (3%)	0
Some people argue that animal research rarely produces benefits to humans. Do you agree that this is likely?^a^						
		**Strongly Agree**	**Agree**	**Uncertain**	**Disagree**	**Strongly Disagree**
	Pediatricians	0	7/43 (16%)	5/43 (12%)	23/43 (54%)	8/43 (19%)
	Nurses/RTs	2/68 (3%)	7/68 (10%)	37/68 (54%)	19/68 (28%)	3/68 (4%)

^a^There was a statistically significant (p < 0.001) difference in response between pediatricians versus nurses/RTs to the question “Some people argue that animal research rarely produces benefits to humans. Do you agree that this is likely?”

**Table 4:** **Healthcare worker expectations for translation to humans from animal research paid for with public funding**

**Statement about an expectation from animal research**						
	**Respondent Group**	**5-20%**	**21-40%**	**41-60%**	**61-80%**	**>80%**
Drugs tested on animals should correctly predict adverse reactions in humans at least what percent of the time?						
	Pediatricians	4/42 (10%)	9/42 (21%)	9/42 (21%)	13/42 (31%)	7/42 (17%)
	Nurses/RTs	3/67 (5%)	7/67 (10%)	14/67 (21%)	18/67 (27%)	25/67 (37%)
Drugs that work well in animal experiments should prove to work in humans for the same disease at least what percent of the time?						
	Pediatricians	5/42 (12%)	11/42 (26%)	8/42 (19%)	14/42 (33%)	4/42 (10%)
	Nurses/RTs	3/67 (5%)	9/67 (13%)	10/67 (15%)	19/67 (28%)	26/67 (39%)
For an animal research result to be credible, a second laboratory should be able to reproduce the results from the first laboratory at least what percent of the time?						
	Pediatricians	0	0	2/42 (5%)	8/42 (19%)	32/42 (76%)
	Nurses/RTs	0	1/67 (2%)	4/67 (6%)	11/67 (16%)	51/67 (76%)
Drugs tested on animals should correctly predict drugs that cause cancer or birth defects in humans at least what percent of the time?						
	Pediatricians	3/42 (7%)	8/42 (19%)	5/42 (12%)	15/42 (36%)	11/42 (26%)
	Nurses/RTs	2/67 (3%)	5/67 (8%)	11/67 (16%)	21/67 (31%)	28/67 (42%)
Drugs that work well in experiments in one type of animal should prove to work well in another closely related animal (for example, in different types of rats) at least what percent of the time?						
	Pediatricians	2/41 (5%)	2/41 (5%)	9/41 (22%)	12/41 (29%)	16/41 (39%)
	Nurses/RTs	0	0	11/67 (16%)	19/67 (28%)	37/67 (55%)
Drugs that work well in animals with stroke, severe infection, cancer, brain or spinal cord injury should work in humans at least what percent of the time?^a^						
	Pediatricians	5/41 (12%)	12/41 (29%)	5/41 (12%)	13/41 (32%)	6/41 (15%)
	Nurses/RTs	4/66 (6%)	4/66 (6%)	9/66 (14%)	23/66 (35%)	26/66 (39%)
Experiments sometimes find a drug beneficial in animals when it is harmful in humans; and, sometimes find a drug harmful in animals when it is beneficial in humans. These misleading animal experiments should occur at most what percent of the time?						
	Pediatricians	24/42 (57%)	12/42 (29%)	5/42 (12%)	1/42 (2%)	0
	Nurses/RTs	38/67 (57%)	19/67 (28%)	6/67 (9%)	3/67 (5%)	1/67 (2%)
Assume drugs studied in animals accurately predict effects in humans less than 20% of the time. If this were true, it would significantly reduce your support for animal research.^a^						
		**Strongly Agree**	**Agree**	**Uncertain**	**Disagree**	**Strongly Disagree**
	Pediatricians	7/42 (17%)	13/42 (31%)	5/42 (12%)	11/42 (26%)	6/42 (14%)
	Nurses/RTs	40/67 (60%)	16/67 (24%)	7/67 (10%)	2/67 (3%)	2/67 (3%)

^a^There was a statistically significant (p < 0.001) difference in response between pediatricians versus nurses/RTs to the two questions: “Drugs that work well in animals with stroke, severe infection, cancer, brain or spinal cord injury should work in humans at least what percent of the time?” and “Assume drugs studied in animals accurately predict effects in humans less than 20% of the time. If this were true, it would significantly reduce your support for animal research.”

### Ethics approval

The study was approved by the health research ethics board 2 of our university (study ID Pro00039590) and return of a survey was considered consent to participate.

### Statistics

The web-based tool (REDCap) allows anonymous survey responses to be collected, and later downloaded into an SPSS database for analysis. The proportions of respondents with different answers were expressed as percentages. The responses of the two predefined groups, pediatricians and pediatric intensive care unit nurses/RTs, were compared using the Chi-square statistic, with *P ≤* .05 after Bonferroni correction for multiple comparisons considered significant.

## Results

### Pediatricians

#### Demographics

Forty-eight responded, but only 44/114 (39%) gave responses to more than the demographic questions. Demographics are given in Table 1.

#### Expectations regarding methodology of AR

The majority of respondents agreed that: anesthetic use should be monitored during surgery (100%), pain should be monitored after this surgery even over-night (91%), and experimenters in a research study should have similar training on the procedures involved (97%) (Table 2). The majority disagreed that it is acceptable: to use less humane methods of euthanasia to reduce costs or improve results (82% or 52% respectively), to use animals when alternatives are available (73%), to do an animal experiment without a systematic literature review (100%), and to do an animal experiment using suboptimal methods (including randomization, blinding, and primary outcome specification) in order to save costs (82-93%). Only a minority of respondents agreed that failed animal models of a disease should continue to be used (30%), or that stressed animals should be used (37%). Finally, the majority agreed that guidelines consistent with these responses should be required for publicly funded AR (95%).

#### Perceptions of human benefits from AR

Most respondents believe that discoveries from AR sometimes or often lead to a treatment for human disease directly (77%) or indirectly (84%), and that researchers sometimes or often claim large benefits from AR (91%) (Table 3). The majority did not agree (84%) with the statement that “AR rarely produces benefits to humans.”

#### Expectations for translation to humans from AR paid for with public funding

The majority of respondents think that drugs tested on animals should correctly predict the following for humans at least 41% of the time: adverse reactions (69% of respondents), disease treatment (62% of respondents), carcinogenicity or teratogenicity (74% of respondents), and treatment of stroke, severe infection, cancer, brain or spinal cord injury (59% of respondents). The majority also expected that replication of AR findings in second laboratories or other strains of the animal should occur at least 61% of the time (95% and 68% of respondents respectively). The majority agreed that misleading (in terms of human benefit and/or harm) animal experiments should occur at most 40% of the time (86% of respondents). Finally, when asked to “assume drugs studied in animals accurately predict effects in humans less than 20% of the time. If this were true, it would significantly reduce your support for animal research”, 40% disagreed (Table 4).

### Pediatric Intensive Care nurses and RTs

#### Demographics

Sixty-nine of 120 (58%) responded; 52 (75%) nurses and 16 (25%) RTs. Demographics are given in Table 1.

#### Expectations regarding methodology of AR

The majority of respondents agreed that: anesthetic use should be monitored during surgery (98%), pain should be monitored after this surgery even over-night (96%), and experimenters in a research study should have similar training on the procedures involved (96%) (Table 2). The majority disagreed that it is acceptable: to use less humane methods of euthanasia to reduce costs or improve results (87% or 81%), to use animals when alternatives are available (88%), to do an animal experiment without a systematic literature review (96%), and to do an animal experiment using suboptimal methods (including randomization, blinding, and primary outcome specification) in order to save costs (87-95%). Only a minority of respondents agreed that failed animal models of a disease should continue to be used (27%), or that stressed animals should be used (19%). Finally, the majority agreed that guidelines consistent with these responses should be required for publicly funded AR (91%).

#### Perceptions of the benefits to humans from AR

Most respondents believe that discoveries from AR sometimes or often lead to a treatment for human disease directly (84%) or indirectly (88%), and that researchers sometimes or often claim large benefits from AR (97%) (Table 3). The majority did not agree (87%) with the statement that “AR rarely produces benefits to humans.”

#### Expectations for translation to humans from AR paid for with public funding

The majority of respondents think that drugs tested on animals should correctly predict the following for humans at least 41% of the time: adverse reactions (85% of respondents), disease treatment (82% of respondents), carcinogenicity or teratogenicity (89% of respondents), and treatment of stroke, severe infection, cancer, brain or spinal cord injury (88% of respondents). The majority also expected that replication of AR findings in second laboratories or other strains of the animal should occur at least 61% of the time (92% and 83% of respondents respectively). The majority agreed that misleading (in terms of human benefit and/or harm) animal experiments should occur at most 40% of the time (84% of respondents). Finally, when asked to “assume drugs studied in animals accurately predict effects in humans less than 20% of the time. If this were true, it would significantly reduce your support for animal research”, only 6% disagreed (Table 4).

### Differences between pediatricians versus nurses/RTs

There were few statistically significant differences. Nurses more often responded that drugs for stroke, severe infection, cancer, brain or spinal cord injury should work in humans. Nurses were more uncertain whether AR “rarely produces benefits to humans”, and would be less supportive of AR if it accurately predicted effects in humans <20% of the time.

## Discussion

There are several important findings from this survey. First, most HCW respondents expect that AR is done with high methodological quality, and that costs and difficulty are not acceptable justifications for lower quality. Most expect that guidelines for AR funded with public money should be consistent with these expectations. Second, most respondents thought that there are either sometimes or often large benefits to humans from AR. Most disagreed that “AR rarely produces benefit to humans.” Third, most respondents expect that AR findings should translate to humans at least 41% of the time, with many expecting this at least 61% of the time. This includes AR findings of adverse events (toxicity), carcinogenicity and teratogenicity, and disease treatments. The majority thought misleading AR results should occur no more often than 20% of the time. If translation from AR to humans was to occur <20% of the time, most would be less supportive of AR. Finally, most respondents expect that AR findings should be reproducible between laboratories and between strains of the same species. There are important implications of these findings for public and HCW acceptance of AR (Table 5).

**Table 5:** **Possible important implications of the findings from this survey**

**Respondents’ expectation**	**Published empirical evidence**	**Possible implication(s)**
AR is done using the best known methods: high standards of animal welfare.^a^	Compatible with recommendations of recent guidelines from the UK, USA, and Canada [63-65]. Studies have found poor reporting of animal welfare, including poor attention to pain control, and not using the most acceptable methods of euthanasia [11,12].	AR may need to be of much higher animal welfare quality in order to maintain public and HCW support.
AR is done using the best known methods: high standards of methodological quality.^b^	Compatible with recommendations of recent guidelines from the UK, US, and Canada [63-65]. Studies have found poor methodological quality of AR in multiple research areas, including after publication of the ARRIVE guidelines [6-12].	AR may need to be of much higher methodological quality in order to maintain public and HCW support.
AR often produces benefit to humans.	Press releases by academic medical centers often promote AR, and most claim relevance to human health without caveats about extrapolating results to people [66]. Of published basic research papers, 0.004% led to the development of a clinically useful class of drugs [67].	Most HCW may not be aware of the literature regarding translation of AR.
AR has high translation rates of findings to humans, including in the areas of toxicology, carcinogenicity, teratogenicity, and therapeutic success.^c^	Translation rates from AR to humans are at best 0-5% in the fields of sepsis, stroke, spinal cord injury, traumatic brain injury, cancer, degenerative brain diseases, acquired immunodeficiency syndrome, asthma, and others [16-32]. Pharmaceutical drug development translation from AR to humans is about 8% [33,34]. Reviews of high impact published AR have found translation rates are at best 1-10% [15-18,67-69].	AR may need to be much better at predicting human responses to drugs and disease in order to maintain public and HCW support.

AR: animal research; HCW: health care workers.

^a^ For example, monitoring and titration of anesthesia, monitoring and titration of pain control even over-night, using the most humane known methods of euthanasia, avoiding stressed animals, and using the fewest number of animals possible.

^b^ For example, performing a systematic literature review to inform study design, using optimal design including randomization and blinding, attention to training of staff, and to choosing models that have shown translation of findings to humans.

^c^ For example, most think translation rate should be over 40%, that misleading results for humans should occur no more than 20% of the time, and that if this was not the case their support for AR would be significantly reduced.

Previous public surveys have generally asked only whether people support AR for human benefit, and not asked people to evaluate the details of their expectations of AR. For example, the Eurobarometer asks “scientists should be allowed to experiment on animals like dogs and monkeys if this can help sort out human health problems”; in 2010, 44% of Europeans responded ‘agree’ and 37% ‘disagree’ [44]. This support for AR was linked with “greater appreciation of the contributions of science to the quality of life” and “an omnipotent vision of science” [45]. In the UK the 2012 Ipsos MORI determined that most (85%) are ‘conditional acceptors’ of AR; people accept AR “so long as it is for medical research purposes”, “for life-threatening diseases”, “so long as there is no unnecessary suffering”, or “where there is no alternative”, considering AR as a “necessary evil” for human benefit [41]. In the United States the 2011 Gallup’s Values and Beliefs survey found that when asked whether medical testing on animals is morally acceptable or morally wrong, 43% (and 54% of young adults 18-29 yr old) responded ‘morally wrong’ [46]. In a survey in Sweden including patients with rheumatoid arthritis and scientific expert members of research ethics boards, most respondents agreed to AR for at least some type of biomedical research. Support was highest for AR into “fatal diseases” (83.1%), and diseases with “insufficient treatment options” (82.1%) [47]. In a UK survey of scientists promoting AR, lay public, and animal welfarists, the support for AR (on a Likert scale of 7) was 5.33 (1.46), 3.57 (1.70), and 1.48 (0.87) respectively. Scientists and lay public supported animal use only for “medical research”, and not for dissection, personal decoration, or entertainment [48]. These surveys suggest people support AR on the understanding that it is necessary to provide significant benefit for humans with severe diseases, and is done to high ethical standards. However, none asked for the amount of detail as in our survey.

Some qualitative research also suggests there is conditional public acceptance of AR based on a utilitarian analysis of costs (to animals) and benefits (to humans) [49,50]. This conditional acceptance is usually based on the assumption that regulation has assured AR is to high animal welfare standards, of high scientific validity and merit (i.e., high quality research, leading to human benefit and cures), and that there are not alternative research methods [49-51]. Scientists understand this role of regulation as leading to societal acceptance of AR, and see regulation as legitimating AR practice [51-53]. However, our survey suggests that this trust in regulation may be misplaced, because regulation does not result in AR that meets HCW expectations for animal welfare, methodological quality, human benefit, or rates of translation to human medicine and cures (Table 5). Moreover, these studies showed that the public is far less accepting of the use of genetically modified animals in research, based on a deontological approach where this AR is seen as ‘wrong’ [49,50]. We did not ask about the common use of genetically modified animals in AR, and therefore may have underestimated HCW expectations of AR.

There are two main explanations for the poor predictive accuracy of AR for humans. First, it is possible that the poor methodological quality of AR has resulted in a biased literature that has led to many human trials based on inappropriate data. Second, it is possible that animal models are not good ‘causal analogical models’; not useful to extrapolate findings to humans because there are major causal disanalogies between species [54,55]. Animal models are based on this reasoning: when an animal model is similar to the human with respect to traits/properties a,b,c [e.g. fever, hypotension, and kidney injury in sepsis], and when the animal model is found to have property d [e.g. response to protein-C treatment], then it is inferred that the human also likely has property d. This ‘causal analogy’ assumes that there are few causal disanalogies: few properties e,f,g that are unique to either the animal or human and that interact causally with the common properties a,b,c. However, animals are evolved complex systems; they have a myriad of interacting modules at hierarchical levels of organization [56]. As a result of this complexity, animals have emergent properties [e.g. animal traits/functions, like property d] that are dependent on initial conditions [e.g. gene expression profiles, the context of the organism, like properties a,b,c, *and* e,f,g]. In complex systems [e.g. animals], very small differences in initial conditions [e.g. properties e,f,g specific to a species/strain] can result in dramatic differences in response to the same perturbation [e.g. drug, treatment, or disease leading to property d] [54-58]. There is much empirical data finding major causal disanalogies between animal species: differences in gene expression at baseline and in response to perturbations, and in disease susceptibilities [59-62]. Thus, complexity science suggests there may be an in principle limitation for AR to predict human responses. Our survey suggests that these competing explanations must be sorted out to determine whether translation can meet public expectations in weighing the costs and benefits of AR.

This study has several limitations. Response rates for pediatricians and nurses/RTs were 39% and 58% respectively; thus we cannot rule out biased participation in the survey. Statements presented needed to be short and concise, and this may have left out important details that would have influenced the understanding of and response to the text. The moderate sample size from one University limits the generalizability of our results. Nevertheless, this is the first survey we are aware of that asks any group not just to consider whether they support AR; rather, to consider in detail the expectations for the methodology and translation of AR. Strengths of this study include the rigorous survey development process, and the inclusion of the most common critiques of the empirical practice of AR. Future study should determine the generalizability of our results.

## Conclusion

We found HCW respondents had high expectations for the methodological quality of AR, and the translation of findings from AR to human responses to drugs and disease. These expectations are far higher than the empirical data show having been achieved. This disconnect between HCW expectations of AR and the empirical reality of AR suggests that if HCW were better informed they would likely withdraw their conditional support of AR. Improved methodological quality is an achievable goal if this is prioritized by researchers, reviewers, editors, and funders. Whether methodologically optimal AR can achieve better human translation to meet HCW expectations is an open question.
